# Diagnostic accuracy of staging of Wilms’ tumour in the era of multislice CT

**DOI:** 10.1186/1470-7330-14-S1-P18

**Published:** 2014-10-09

**Authors:** S Kembhavi, S Qureshi, M Ramadwar, P Popat, G Chinnaswamy, S Laskar

**Affiliations:** 1 Tata Memorial Centre, Mumbai, India

## Aim

To assess the diagnostic accuracy of CT in local staging of Wilms’ Tumour.

## Method

Audit of radiology reports (16 slice CT), surgical notes and histopathological reports in 24 cases of unilateral non-metastatic Wilms’ tumour (2012 to 2014).

## Results

24 patients were eligible. 12 boys, 12 girls, age range of 1-10 years (mean 3.9). 6 patients underwent upfront surgery (Group A) while 18 patients received 4 weeks of chemotherapy (Group B). The post chemotherapy scans were compared to gold standard in the latter group.

Renal vein involvement: Present in 8 patients (all group B), CT had 100% sensitivity, 90% specificity, NPV 100%.

Renal sinus involvement: Present in 14 patients (4 group A, 10 group B). Sensitivity and specificity of CT was 25%, 100% for group A and 90%, 50% for group B.

Renal pelvis involvement: Present in 8 patients (1 group A, 7 group B). Sensitivity and specificity of CT was 71.4%, 81.8% for group B and specificity of 100% for group A.

Renal Capsular involvement (but not necessarily the margin) was present in 6 patients (2 group A, 4 group B). Sensitivity and specificity of CT was 50%, 100% for group A and 42.8%, 75% for group B.

Overall, CT stage matched histopathological stage in 4/6 patients in group A and in 12/18 patients in group B (66.6% in both groups).

## Conclusion

CT staging has higher specificity in upfront surgery, probably because of the smaller tumour size. The sensitivity of CT staging with regards to renal vein, sinus and pelvic involvement is better than renal capsular involvement, where CT tends to over-stage disease in larger tumours.

